# The Perception and Attitude of Farmers toward Domestic Waste Classifications: A Case Study on Wusheng County, Sichuan Province, China

**DOI:** 10.3390/ijerph192013499

**Published:** 2022-10-19

**Authors:** Xuxi Wang, Jing Tan

**Affiliations:** 1 Policy Research Office, Sichuan Institute of Urban and Rural Construction, Chengdu 610041, China; 2 School of Public Policy and Administration, Chongqing University, Chongqing 400044, China; 3 Center for Public Economics & Public Policy, Chongqing University, Chongqing 400044, China

**Keywords:** rural domestic waste, classification behavior, perception, influencing factors, farmers

## Abstract

The effective treatment of rural domestic waste is the key to solving rural environmental pollution and realizing rural ecological revitalization. Giving full play to the main role of farmers’ domestic waste classification can improve the efficiency and effect of domestic waste treatment. To explore the key factors affecting the farmers’ perception, attitude, and behavior of domestic waste classification, this study with 318 farmers in Sichuan Province as the research object, the research framework of domestic waste classification behavior was constructed based on the theory of planned behavior, and then, the logistic regression model was used for the empirical test. The results show that the farmers’ education levels, subjective norms, relatives’ and neighbors’ views on waste classification, farmers’ awareness regarding the negative environmental impacts caused by waste, farmers’ private benefits, and farmers’ views on the waste management ability of local governments are significantly positively correlated with the classification behavior of farmers’ domestic waste. The distance between farmers’ houses and waste collection points is significantly negatively correlated with the classification behavior of farmers’ domestic waste. This paper provides a certain theoretical reference for realizing the reduction, resourcization, and positive development of rural domestic waste management in China.

## 1. Introduction

In recent years, the income of rural residents in China has significantly increased; however, with these improvements and lifestyle changes, the amount of rural domestic waste has increased sharply. For many areas, the issue of “waste surrounding villages” has affected the ecological environment of rural human settlements and the health of rural residents. Rural waste is not only a huge quantity but also often presents the characteristics of random mixing and dumping. The traditional extensive mixed treatment has been unable to achieve the effective management of rural domestic waste and the fundamental improvement of the ecological environment. Domestic waste classification is the fundamental way to solve the pollution of rural domestic waste and improve the long-term operation mechanism of rural domestic waste treatment [1,2]. Classified governance is a key task in building new green countryside and an important measure to improve the well-being of rural residents.

However, the current research on the behavior of domestic waste classification is mainly concentrated in cities [3,4], and there is a lack of micro-empirical research at the farmer level. However, the obstacles faced by domestic waste disposal in rural areas are greater than those in urban areas [5]. Compared with urban residents, the rural population has scattered domestic arrangements, imperfect waste treatment facilities, and inadequate waste classification management, which hinder the development of domestic waste classification. Therefore, it is particularly important to explore the main influencing factors and mechanism of classification behavior of farmers’ domestic waste (FDW) and then put forward corresponding policy suggestions to improve the participation rate of FDW classification and the efficiency of rural domestic waste treatment.

The participation of farmers is an indispensable link for improving the efficiency of rural domestic waste classification [6]. Farmers are not only the producers and victims of rural domestic waste but also the direct beneficiaries of governance effects. They have the endogenous power to participate voluntarily in waste classification behavior [7,8]. Giving full play to the farmer’s main role in domestic waste classification can improve the efficiency and effect of domestic waste management to improve the rural living environment and comprehensively promote rural economic and social development. However, few kinds of literature on rural waste classification mainly study the aspects of equipment and facilities, capital investment, environmental policies, etc., while few studies involve the attitude and emotional factors of farmers themselves, and the problem of low participation of governance subjects has always existed.

The Theory of Planned Behavior (TPB) is an important theory in the field of behavioral science, which is mainly used to study the behavioral motivation of individuals and can help researchers understand how individuals change their behavior patterns. TPB posits that the generation of environmental behavior comes from an individual’s own expectations and welfare, proposing that behavioral attitudes (BAs), subjective norms (SNs), and perceived behavioral control jointly determine an individual’s environmental behavior [7]. Currently, The TPB has consistently demonstrated its utility and efficacy in explaining recycling in European countries and domestic waste classification in various settings and contexts, including LMICs in the Eastern Mediterranean (Turkey and Greece), Africa (South Africa and Ghana), and Asia (India, Iran, Saudi Arabia, and China) [9,10,11]. Therefore, introducing the TPB into FDW classification management behavior research, from the aspects of rural residents itself, to explore the internal factors influencing the desire and behavior of its classification can improve the enthusiasm for FDW classification management. It is helpful to establish and improve the system of FDW management in villages and solve the problem of FDW management from the root.

Sichuan has always been a major agricultural province in China. As of 2021, Sichuan had a permanent resident population of 83.72 million, of which 38.7 million—or about 46.2 percent—were agricultural residents. Due to the complex mountainous environment of Sichuan Province, rural areas in Sichuan are characterized by a low domestic density, extremely dispersed settlements, and large regional differences, which makes waste management and disposal in these areas more difficult. Based on questionnaire survey data at the farmer level and the TPB framework, this study constructed an FDW classification behavior model to explore the impact of farmers’ BAs, SNs, individual perceptions, and awareness regarding rural domestic waste classification behavior to provide a theoretical reference for the adjustment and improvement of rural domestic waste classification management policy.

The rest of the paper is organized as follows: Section 2 details the main structure of the TPB model. Section 3 provides the data, variables, and methods of our study. Section 4 presents the empirical results. Section 5 is for discussion. Section 6 ends the paper by proposing some conclusions.

## 2. Literature Review

TPB framework has a very high reference value for the research of predicting and explaining behavior relationships [12]. TPB explains the general decision-making process of individual behavior from the perspective of subjectively perceived information related to behavior, starting from the theory of expected value [13]. TPB can be seen in four stages: In the first stage, it is believed that in addition to the influence of behavioral intention, actual behavior is also restricted by control conditions such as ability and resources. Among them, perceptual and behavioral control will be correlated with behavior as a subjective proxy variable of actual control conditions. In the third stage, the salient beliefs of individuals affect behavioral attitudes, subjective norms, and perceived behavioral control. In the fourth stage, individuals have many beliefs related to behavior, and only a part of them can be acquired subjectively under a specific time and environment. These accessible beliefs are called salient beliefs, and all other factors must indirectly influence these salient beliefs [14,15,16].

The application of this theory requires the analysis of the three main structures:

(1) BA. BA is an assessment of how much an individual likes or dislikes a particular behavior, which is derived from accessible behavioral beliefs, including belief strength and evaluation of the results of the behavior. BA is generally considered to be the most powerful predictor of behavioral intention [17,18], but which variable should be selected to measure behavioral attitude more accurately and effectively is of more interest to the academic community. Some scholars believe that TPB should emphasize not only instrumental attitude, that is, it should only be judged by whether it has value, but also measure emotional attitude, that is, whether it is liked or not [19]. In recent years, rural domestic waste management has gradually become the focus of society. Farmers’ evaluation of domestic waste classification will directly affect the intention of domestic waste classification;
(2) SN. SNs mainly reflect the social pressure of individuals by normative belief (the expectation that important others or teams support or oppose individual behavior) and compliance motivation (the degree to which individuals comply with the expectation or pressure). In most studies, SNs have the weakest effect on intention [20]. Cialdini [21] proposed that norms are a multidimensional concept, which should be divided into personal norms (what individuals think they should do), demonstrative norms (what significant others think individuals should do), and prescriptive norms (what significant others think individuals should do). The countryside belongs to a typical “acquaintance society” network. If farmers believe that the classification of domestic waste conforms to the beliefs of family members or neighbors, they are more likely to classify domestic waste under pressure.
(3) Perceptual behavioral control refers to the self-efficacy of an individual to complete a specific behavior (the perception of whether he is capable of achieving the behavior) and the ability to control the behavior [22], which is mainly influenced by the belief in control and the intensity of perception [13]. For domestic waste classification, farmers believe that the fewer expected constraints on their behavior, the stronger the corresponding classification behavior [23,24,25].

**Figure 1:**
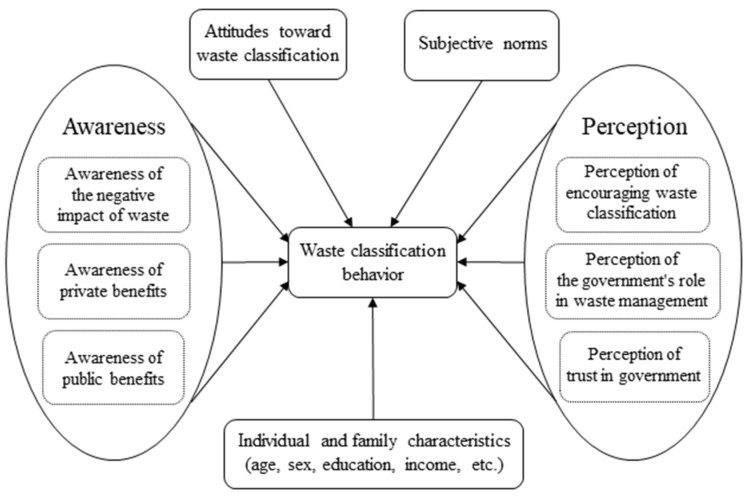
Theoretical analysis framework.

Compared with other relevant theoretical models, such as the normative activation theory proposed by Schwartz [25] and the self-regulation theory proposed by Bagozzi [26], the TPB model is a more open model, which shows an effective variable structure in specific analysis. If other variables can play a significant role in predicting behavioral variability, they can be included in the theoretical model [27]. Therefore, some scholars added other variables to predict human behavior [28], while some scholars removed perceptual and behavioral control variables due to difficulties in observation and measurement [29]. For example, Zhang et al. (2017) measured the perception level of individuals on waste classification [30], and Nguyen et al. (2019) added individual perception and awareness variables into their analysis [31]. Different from other existing literature, the innovation of this study is to select individual perception and awareness as additional determinants and explore FDW classification behavior based on the extended TPB framework. According to the ideas of [32,33], the theoretical analysis frame diagram was constructed (Figure 1).

## 3. Materials and Methods

### 3.1. Sample Data

The research data were obtained from a field questionnaire survey conducted in Wusheng County, Guang’an City, Sichuan Province, from April to May 2021. Four villages—Baiping Village, Gaodong Village, Hongmeixin Village, and Lushan Village—were selected as the sample villages. The equal probability random sampling method was used to select the survey sample. On this basis, a questionnaire survey and telephone interview were used to analyze the sample population. The questionnaire was designed according to the field survey results and previous research results on the factors influencing FDW classification. The questionnaire was divided into five parts and scored on a 5-point Likert scale. It takes 0.5 h on average to complete each questionnaire. In total, 326 questionnaires were obtained, of which 318 were valid (had complete information), reflecting an effective rate of 97.55%.

### 3.2. Variables

BA, SN, individual perception, and awareness are all conceptual factors, namely latent variables, which cannot be directly observed and measured. They must be obtained through indirect inferences and estimation through other specific observable indicators. In this paper, based on the interpretation of Ajzen’s TPB and relevant indicators in previous research [34], the measurement scale is finally determined according to the actual characteristics of rural domestic waste classification. The specific measurement items are shown in Table 1. The measurement items of latent variables are shown in Table 2.

### 3.3. Econometric Model

This paper studies the FDW classification behavior, including two situations: no waste classification and waste classification. According to the theoretical analysis framework, the FDW classification behavior is mainly affected by BA, SN, individual perception and awareness, and individual and family characteristics. Therefore, this study takes whether farmers exhibit actual waste classification behavior as the dependent variable, namely the 0–1 dependent variable (waste classification behavior, *y* = 1; no waste classification behavior, *y* = 0), and construct a binary logistic model to explore the influencing factors of FDW classification behavior. The formula is as follows:

(1) $$P(y=1|(x_{1},x_{2},\ldots ,x_{m})=\frac{\exp(\alpha +\sum_{i=1}^{n}\beta_{i}x_{i})}{1+\exp(\alpha +\sum_{i=1}^{n}\beta_{i}x_{i})}$$

where *P_i_* is the probability that farmers exhibit waste classification behavior; *β_i_* (*i* = 1, 2, …, *n*) is the regression coefficient of influencing factors; *i* is the number of influencing factors; *n* is the total number of influencing factors; *x_i_* (*i* = 1, 2, …, *n*) is the independent variable; *α* is a constant term; and *ε* is the random error.

After transformation, the probability occurrence ratio of *y* = 1 and *y* = 0 can be obtained in the form of

(2) $$P_{i}=\alpha +\sum_{i=1}^{n}\beta_{i}x_{i}+\varepsilon_{i}$$

where *β_i_* is the change in the ratio of the natural logarithm of the probability that the farmer exhibits waste classification behavior to the probability that they do not exhibit waste classification behavior when the independent variable *x_i_* changes by one unit.

## 4. Results

This study used the Harman single-factor method to verify the common method deviation problem. The specific operation process involved conducting an unrotated principal component factor analysis on all items of the scale used to verify whether there was a common deviation problem. The results show that the variance contribution rate of the first factor is 23%, so there is no common method bias.

### 4.1. Social and Economic Characteristics of the Interviewed Farmers

The average age of the respondents is 61.75; there are more females, accounting for 62% of the total sample (Table 3). Moreover, the educational level of the interviewed farmers is low, their average years of education is 5.46, and the average annual cash income of the families is 53,200 yuan. In addition, in the sample, the houses are far away from trash cans, with an average distance of 1.26 km.

### 4.2. Attitude towards Waste Classification

The surveyed farmers were asked to use ‘useful’, ‘necessary’, and ‘interested’ to indicate their attitude towards domestic waste classification. In the overall sample population, the average value of BA is 3.77 (Table 4). Among the surveyed farmers, more than 50% agree that ‘waste classification is useful’, and only 5.66% disagree or strongly disagree that ‘waste classification is useful’. More than 60% of the farmers think it is very necessary or necessary to separate waste, and only 1.26% of farmers think it is very unnecessary. More than 50% of the farmers showed interest in waste classification, while 13.21% showed no interest in waste classification (Table 5).

### 4.3. SN

**Figure 2:**
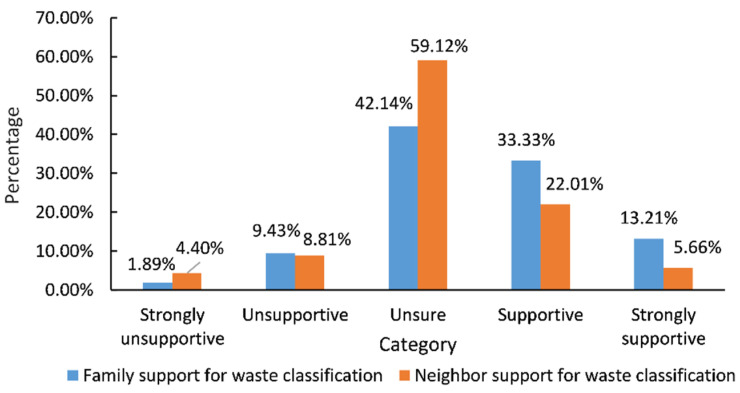
Support of family and neighbors for waste classification.

In the total sample population, the average SN of the farmers is 3.31 (Table 4). From Figure 2, it can be concluded that the support of the family is more important for waste classification measures than that of neighbors.

### 4.4. Awareness of Waste Classification

**Figure 3:**
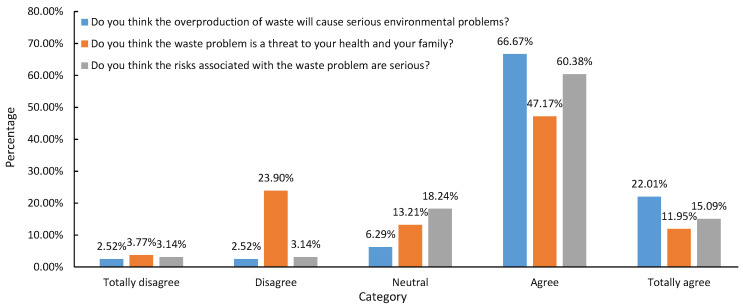
Farmers’ awareness of the negative impact of domestic waste.

Through ‘the seriousness of the impact of waste on the local environment’, ‘the threat of waste to family health’, ‘the risk of waste’, and other related issues, the farmers’ awareness of the negative impact of waste could be understood. According to Table 4, farmers have a particular awareness of the negative effects of waste, with an average value of 3.75. Among the surveyed farmers, nearly 90% believe that excessive waste production will cause serious environmental problems, and only 5.04% believe that excessive waste production will not cause serious environmental problems. In total, 59.12% believe that the waste problem poses a threat to their health. Moreover, 75.47% of the farmers also considered the risks associated with waste problems to be serious (Figure 3).

**Figure 4:**
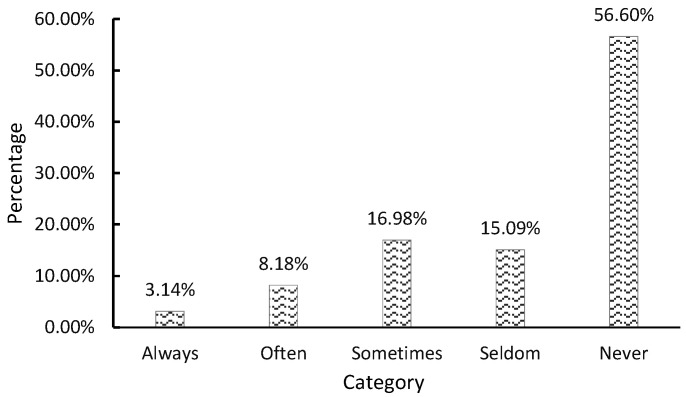
Farmers’ behavior in terms of private benefits.

**Figure 5:**
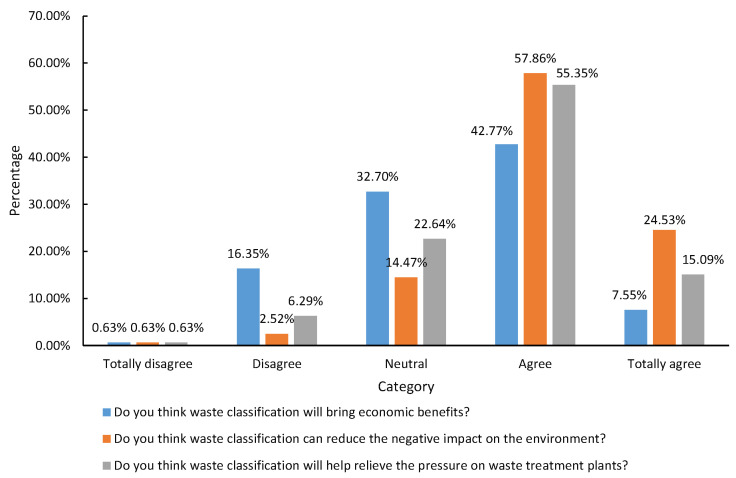
Farmers’ behavior in terms of public benefits.

Further, farmers were also asked about waste classification’s positive effects and benefits. This study divides these benefits into two categories: (1) Private benefits: The economic income reaped by waste classification. (2) Public benefits: Waste classification eliminates the negative impact on the environment and reduces the pressure on waste disposal sites. According to Table 4, the average value of private benefits for farmers is 2.63, which is significantly lower than the average value of public benefits for farmers (3.91). In terms of private benefits, 50.32% of the surveyed farmers believe that waste classification will lead to some economic benefits, and 16.98% believe that waste classification will not bring about any economic benefits. In total, 56.60% of the farmers never receive economic benefits from waste classification, while the farmers who often or always reap economic benefits from the waste classification are fewer, accounting for 8.18% and 3.14% of the total sample, respectively (Figure 4). In terms of public benefits, 82.39% of the surveyed farmers believe that waste classification can reduce the negative impact of waste on the environment, only 3.15% of the farmers believe that waste classification cannot reduce the negative impact of waste on the environment, and 70.44% of the farmers believe that waste classification can reduce the pressure on waste disposal plants (Figure 5). Only 6.92% of farmers believe that waste classification did not impact waste disposal plants.

### 4.5. Perception

**Figure 6:**
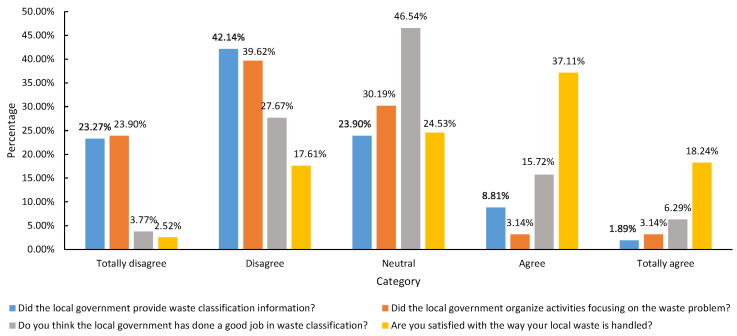
Farmers’ perceptions of the role of local authorities in waste management.

According to Table 4, the average value of farmers’ perception regarding encouraging waste classification is 3.92, which indicates that most farmers encourage waste classification, believing that this classification can make the environment cleaner and reduce the cost of waste disposal. In this study, farmers’ perceptions of the government’s provision of waste classification information, waste classification management, and waste disposal methods are generally reflected in farmers’ views on the role of the government in waste management. According to Table 4, the average value of farmers’ understanding of the government’s waste management function is 2.73, which indicates that the government’s waste management ability needs to improve. Less than 10% of the surveyed farmers believe that the local government often provides information on waste classification. Most farmers believe that the local government never or rarely carries out activities focusing on the waste problem; 46.54% of farmers agree with the government’s waste classification management, and only 3.77% of farmers agree with it very much (Figure 6). In addition, this study investigated farmers’ trust in the waste management policies of the local government and their trust in the waste management ability of the local government, with an average value of 3.19 (Table 4), indicating that farmers have a certain degree of trust in the waste management ability of their local governments.

### 4.6. Waste Classification Behavior

**Figure 7:**
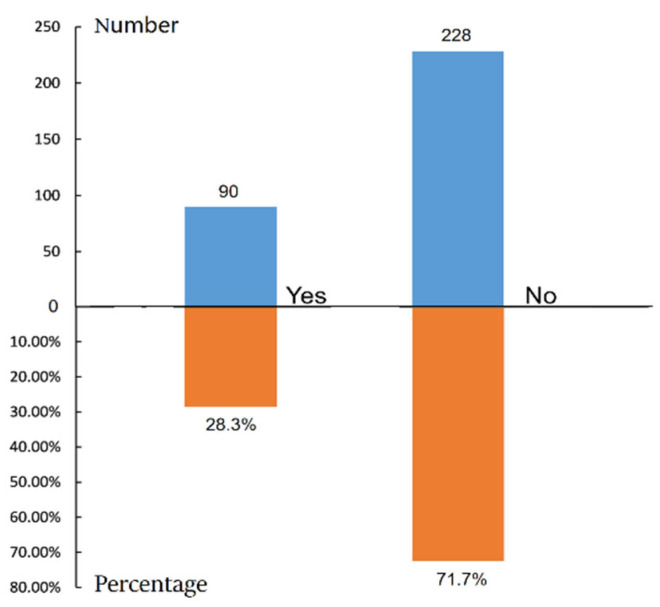
Farmers’ waste classification behavior.

The classification of domestic waste is essential for implementing a sustainable waste management system. However, due to the relatively short duration since the implementation of waste classification initiatives in rural China, farmers’ participation in waste classification is not high, and their action power is insufficient. According to the survey of domestic waste classification behavior, 71.70% of the farmers did not classify waste in their daily life, and only 28.30% of the farmers practiced waste classification (Figure 7). It is clear that only a small proportion of the rural population practiced waste classification. There is, therefore, a need for evidence-based interventions that are acceptable to farmers—in a cost-effective, context-specific, and participatory manner—in rural areas [35].

### 4.7. Econometric Model Results

Before constructing the model, a collinearity analysis is conducted on all independent variables. It is found that the variance inflation factors (VIFs) of the 13 independent variables selected are all less than 10, indicating no serious multicollinearity; therefore, the model can be constructed [36].

Based on the survey data and the theoretical analysis framework of this study, a binary logistic regression model was constructed to explore the effects of BA, SN, individual perception and awareness, and individual and family characteristics on FDW classification behavior. The Nagelkerke R2 value of the model is 0.724, indicating that the fitting degree of the model is good.

The influence of individual characteristic variables of farmers on domestic waste classification behavior is as follows: according to Table 6, age, gender, and annual family income have no significant influence on FDW classification behavior; the education level of farmers has a significant positive correlation with their domestic waste classification behavior at a significance level of 10%. The higher the education level, the more likely the individuals are to separate domestic waste. The distance from the house to the waste collection point has a significant negative correlation with the domestic waste classification behavior at the 5% significance level. The closer the house is to the waste collection point, the more likely they are to separate domestic waste.

The impact of the BA variable on FDW classification behavior is as follows: according to Table 6, the BA of farmers has a positive effect on their domestic waste classification behavior, which is significant at the 5% level. That is, if the BA score of farmers increases by one unit, their probability of separating domestic waste will increase by 41.1%.

The influence of SN variables on FDW classification behavior is as follows. ‘The opinions of relatives and neighbors on domestic waste classification’ has a significant positive influence on FDW classification behavior, which is significant at the 10% level (Table 6). That is, the more the relatives and neighbors support FDW classification behavior, the greater the probability of domestic waste classification.

The impact of farmers’ awareness variables on FDW classification behavior is as follows: the farmers’ awareness of the negative effects of domestic waste on the environment is significantly positively correlated with FDW classification behavior, which is significant at a 5% level. That is, the more the individual recognizes the negative impact of domestic waste on the environment, the greater the probability of domestic waste classification. The private benefits of farmers are also positively correlated with FDW classification behavior, with a correlation coefficient of 4.646, which is significant at the 1% level. That is, if farmers believe that domestic waste classification generates economic value for them, the possibility of domestic waste classification is greater.

The farmers’ perception variables on FDW classification are as follows. According to Table 6, farmers’ perceptions of encouraging waste classification and farmers’ trust in the government have no significant impact on FDW classification, while farmers’ perceptions of the local government’s waste management ability have a significant positive impact on FDW classification, which is significant at the 10% level. That is, farmers believe that the stronger the government’s ability for waste management, the more likely it is for farmers to carry out domestic waste classification.

## 5. Discussion and Policy Implications

This study investigated the influence of farmers’ BA, SN, individual perception, and awareness regarding FDW classification behavior. A TPB model that integrates planned behavior, norms, and expectations is established, which deepens our current understanding of the expectation mechanism and the resulting behavioral intentions that influence waste classification. The TPB model provides an effective framework for identifying the primary determinants of waste classification behavior.

The research shows that the distance between individuals’ houses and the waste collection points is significantly negatively correlated with FDW classification behavior. This means that the farther away the waste collection point is, the more inconvenient it is for rural residents to sort rural waste and protect the environment [37]. In addition, this results in a higher opportunity cost of taking the time to sort the waste and therefore dispose of it correctly everywhere [38,39]. This finding is consistent with the conclusion of the authors [35] that the distance between the farm household and the refuse collection point plays a major role in whether a person handles waste anywhere or within a prescribed area. This, to some extent, reflects that the waste classification infrastructure impacts the waste classification of farmers. Inadequate refuse collection facilities have a great impact on residents. At present, there are two major problems in the FDW and recycling system in rural areas of Sichuan Province. First, the traditional two-color waste cans (recyclable and non-recyclable) are dominant in most rural areas, and the four-color waste cans with clear labels are insufficient. Second, waste classification is still mixed transportation. There is no effective classification transportation system. Therefore, the government should strengthen the supply of infrastructure for domestic waste classification; rationally arrange collection points for domestic waste classification by taking into account the characteristics of rural population structure, population size, road setting, housing layout, etc.; and arrange easily identifiable, simple, and convenient waste classification supporting facilities.

The results show that the attitude of farmers toward waste classification and the attitude of family and neighbors toward domestic waste classification is significantly positively correlated with their waste classification behavior. This result was also observed in a previous study on the domestic waste classification behavior of urban residents [40]. Shi et al. [3] investigated waste classification intention and domestic waste classification behavior in China’s urban–rural integration areas and found that among perceived behavioral control, SNs, and consequence awareness, the attitude has the greatest impact on waste classification intention. Although the impact of attitude on waste classification intention and domestic waste classification behavior varies in space, the importance of attitude on waste classification intention and domestic waste classification behavior is significantly higher in the United States [41], Iran [29], and Malaysia [42] was consistently confirmed. Therefore, in the implementation process of waste classification, an accurate multi-subject cooperation mode should be selected. In addition, the rural social structure, social relationships, blood relations, local culture, and other factors also have an impact on the domestic waste classification mechanism [3]. In the process of waste classification, rural blood relations or social acquaintance can be used to encourage villagers to implement domestic waste classification.

This study shows that the private benefits of farmers are positively correlated with their waste classification behavior. That is, if farmers believe that domestic waste classification can generate economic value for them, the more likely they are to carry out domestic waste classification. This is slightly different from the results of some studies on the influencing factors of urban waste classification. For example, the empirical results of [38] found that the benefits were not significant. The possible reason is the income difference between urban and rural areas, which magnifies the effect of benefits in rural areas. Therefore, the government should implement a reward and punishment system for waste classification through collective decision making to encourage farmers to engage in this behavior. For example, a waste classification credits exchange activity, with the concept of ‘classification for credits, credits exchange, and benefits from conversion’ could be implemented. A series of measures, such as incentives, should be given to farmers who actively practice waste classification, which can improve their enthusiasm and participation.

The results show that there is a significant positive correlation between the farmers’ perception of the waste management ability of the local government and their waste classification behavior. Herein, the survey found that farmers think that the local government’s propaganda on waste classification and the organization of waste classification-related activities are insufficient. Similarly, Min T et al. also stressed that waste classification needs strong publicity from the government [43]. At the beginning of the implementation of waste classification, people need to change their living habits. Therefore, the government should establish a comprehensive coordination mechanism for domestic waste classification, carry out extensive publicity and training on domestic waste classification, enhance farmers’ green consumption ideas and environmental protection awareness, and guide farmers in conducting correct waste classification.

This study combines the TPB and the Logistic Regression model. The TPB is based on the perception process; that is, attitude and willingness promote the occurrence of behavior. However, in the process of actual behavior, the interference of uncertain factors will lead to the inconsistency between behavior and willingness, which will affect the significance of the results and thus affect the research conclusion. In the subsequent research, the contradiction between willingness and behavior will be further explored so as to supplement and enrich the related research on the classification and management of rural residents’ domestic waste.

## 6. Conclusions

In recent years, due to the continued development of the rural economy and the change in rural residents’ lifestyles, the output of rural domestic waste has increased significantly. Due to the lack of waste management facilities and appropriate waste management methods in rural areas, the poor management of domestic waste has led to the destruction of the rural ecological environment and had negative impacts on the health of rural residents. The classification of FDW is crucial for improving the rural domestic environment. The perception and attitude of farmers regarding waste classification directly affect the effectiveness of waste classification, which requires increased research attention. In view of this, this study took the Baiping-Feilong Rural Revitalization Demonstration Area in Sichuan Province as an example and, based on the TPB framework; logistic regression was used to analyze the effects of the BA, SN, individual perception, and awareness of farmers on FDW classification behavior. The results show that the education level of farmers, the distance between farmers; houses, and waste collection points, farmers’ attitudes towards waste classification, SN, farmers’ awareness of the negative environmental impact of domestic waste, farmers’ private benefits, and farmers’ perception regarding the capacity of the local government’s waste management are the key factors affecting FDW classification behavior. The regression coefficients of the farmers’ education levels, farmers’ attitudes toward waste classification, SN, farmers’ awareness regarding the negative environmental impact of domestic waste, farmers’ private benefits, and farmers’ perceptions of the local government’s waste management ability were 0.035, 0.344, 0.153, 0.719, 4.646, and 0.868, respectively, and they are significantly positively correlated with FDW classification behavior. The private benefits of farmers had the strongest impact on the FDW classification behavior, and the distance from their houses to the waste collection points was significantly negatively correlated with the FDW classification behavior, with a regression coefficient of −0.195. The results of this study have certain reference significance for the promotion and planning of waste classification in rural China and also provide practical perspectives for researchers and policymakers in other countries.

## Figures and Tables

**Table 1:** Questionnaire composition and measurement indicators.

Variables	Factors	Description of Measurement Indicators
Individual and family characteristics	Age	Age (year)
	Gender	Gender (0 = male, 1 = female)
	Education	Years of education (years)
	Income	Domestic cash income (ten thousand yuan)
	Distance	Distance between waste collection point and home (within 1 = 0.5 km; 2 = 1 km; 3 = 1.5 km; 4 = 2 km; 5 = more than 2 km)
BA	-	5-point Likert scale
SN	-	
Awareness	Negative effects of waste (A1)	
	Private benefits (A2)	
	Public benefits (A3)	
Perception	Perception of encouraging waste classification (P1)	
	Perception of the government’s Waste management capability (P2)	
	Perception of trust in government (P3)	
Behavior	Waste classification behavior	Waste classification in daily life (0 = no,1 = yes)

**Table 2:** Attitude, awareness, perception, and other related measurement items.

Latent Variables	Subindicator (Variable)	Expected Sign	Measured Items
BA	Degree of usefulness	+	Do you think it is useful to carry out waste classification? ^a^
	Degree of necessity		Do you think it is necessary to separate waste? ^b^
	Degree of interest		Are you interested in waste classification? ^c^
SN	Family’s SN	+	Does your family support your waste classification? ^d^
	Neighbor’s SN		Do your neighbors support your waste classification? ^d^
A1	Pollute the environment	+	You think the overproduction of waste will cause serious environmental problems. ^a^
	Threat to health		You think the waste problem is a threat to your health and your family. ^a^
	Severity of related risks		You think the risks associated with the waste problem are serious. ^a^
A2	Economic benefits	+	You think waste classification will lead to economic benefits. ^a^
	Degree of profit		Have you ever benefited financially from waste classification? ^e^
A3	Reduce the level of environmental impact	+	You think that waste classification can reduce the negative impact on the environment. ^a^
	relieve the pressure on waste disposal plants		Do you think that waste classification helps to relieve the pressure on waste disposal plants? ^a^
P1	Make the surrounding environment cleaner	+	Do you think waste classification will make the surrounding environment cleaner? ^a^
	Reduce the cost of waste disposal		You think waste classification helps reduce the cost of waste disposal. ^a^
P2	Government provide information	+	Will the local government provide waste classification information? ^e^
	Government job satisfaction		Do you think the local government has done a good job in waste classification? ^a^
	Satisfaction with waste disposal methods		Are you satisfied with the way your local waste is handled? ^f^
	Government organize activities		Will the local government organize activities focusing on the waste problem? ^e^
P3	Faith in the competence of government	+	Do you trust the local government’s waste management capability and policy? ^g^
	Faith in the government		Do you have confidence in the local government in waste classification? ^a^

^a^: 1 = Totally disagree; 2 = Disagree; 3 = Neutral; 4 = Agree; 5 = Totally agree; ^b^: 1 = Totally unnecessary; 2 = Unnecessary; 3 = Neutral; 4 = Necessary: 5 = Totally necessary; ^c^: 1 = Totally disinterested; 2 = Disinterested; 3 = Neutral; 4 = Interested: 5 = Totally interested; ^d^: 1 = Strongly unsupportive; 2 = Unsupportive; 3 = Unsure; 4 = Supportive; 5 = Strongly supportive; ^e^: 1 = Never; 2 = Seldom; 3 = Sometimes; 4 = Often; 5 = Always; ^f^: 1 = Very unsatisfied; 2 = Unsatisfied; 3 = Neutral; 4 = Satisfied; 5 = Very satisfied; ^g^: 1 = Great disbelief; 2 = Disbelief; 3 = Neutral; 4 = Belief; 5 = Great belief.

**Table 3:** Individual and family characteristics of the interviewed farmers.

Variables	Average	Standard Deviation
Age	61.75	13.13
Gender	0.62	0.49
Education	5.46	3.50
Income	5.33	2.73
Distance	1.26	0.68

**Table 4:** Statistical characteristics of farmers’ attitudes toward waste classification, awareness, perception, and other indicators.

Latent variables	Average	Standard Deviation	Skewness	Kurtosis
BA	3.77	0.82	−0.702	0.584
SN	3.31	0.73	−0.257	1.160
A1	3.75	0.70	−1.223	1.387
A2	2.63	0.76	0.725	0.545
A3	3.91	0.69	−0.746	1.674
P1	3.92	0.68	−0.905	1.192
P2	2.73	0.73	0.652	0.664
P3	3.19	0.81	−0.109	−0.048

**Table 5:** Famers’ attitudes toward waste classification.

Category	Do You Think It Is Useful to Carry out Waste Classification?	Category	Do You Think It Is Necessary to Carry out Waste Classification?	Category	Are You Interested in Waste Classification?
Totally disagree	1.26%	Totally unnecessary	1.26%	Totally disinterested	1.89%
Disagree	4.40%	Unnecessary	15.09%	Disinterested	11.32%
Neutral	13.21%	Neutral	16.35%	Neutral	35.22%
Agree	50.31%	Necessary	44.65%	Interested	35.85%
Totally agree	30.82%	Totally necessary	22.64%	Totally interested	15.72%

**Table 6:** Binary logistic regression analysis of farmers’ waste classification behavior.

Factors	Regression Coefficient	S.E	Wald	Significance Level	Exp (*β*)
Age	0.016	0.019	0.701	0.402	1.016
Gender	−0.195	0.496	0.154	0.694	0.823
Education	0.035	0.030	1.360	0.062 *	0.966
Income	0.024	0.029	0.675	0.406	1.025
Distance	−0.195	0.142	1.885	0.042 **	1.216
BA	0.344	0.419	0.675	0.041 **	1.411
SN	0.154	0.456	0.113	0.074 *	1.166
A1	0.719	0.381	3.554	0.059 *	0.487
A2	4.646	0.573	66.787	0.000 ***	104.157
A3	0.241	0.465	0.269	0.604	1.273
P1	0.550	0.495	0.268	0.577	0.577
P2	0.868	0.542	3.689	0.055 *	0.420
P3	0.425	0.442	0.926	0.336	1.530
Constant	−11.816 ***	2.978	15.740	0.000	0000

Note: *, **, and *** indicate significance at 0.1, 0.05, and 0.01 levels, respectively. Calculated by IBM SPSS Statistics for Windows, version 24 (IBM Corp., Armonk, NY, USA).

## Data Availability

Not applicable.
